# The new normal: A student's lived experiences during the COVID‐19 pandemic

**DOI:** 10.1111/jora.13049

**Published:** 2024-12-12

**Authors:** Marina Francis

**Affiliations:** ^1^ University of Maryland College Park Maryland USA

## INTRODUCTION

In this commentary I discuss how COVID‐19 impacted my life during and after high school, as well as how I believe the pandemic has affected and will continue to affect my generation as a whole. I focus on the sudden feelings of isolation the pandemic caused, and how those impacted my daily life and mental health. Additionally, I expand on the long‐term effects of the pandemic that we still see today, and how legislation and discussions regarding these topics could benefit the youth.

## COMMENTARY TEXT

COVID‐19 had a profound impact on my high school experience. As someone who graduated in 2021, the pandemic mostly affected my junior and senior year of high school, so I was 18 at the time. Toward the end of my junior year is when everything started to shut down; I remember getting a notification in March that our spring break was extended for another 2 weeks. At first, I was super happy about this, and the sentiment was the same among my peers. Then more and more news stories started coming out about how severe the pandemic was and how it was not safe to reopen, and eventually our school told us that we would not be coming back in‐person for the remainder of the year. All my classes were moved to Google Classroom, and all we had to do was prepare for advanced placement exams and complete some assignments here and there. They even told us that our final grades would not be any lower than the ones we had prior to spring break as a curtesy. I was ecstatic at the time as it felt like my summer had started early. However, everything got boring very quickly as we were not allowed to leave the house unless it was for something necessary, like going to the grocery store. I missed seeing my friends and being able to interact with people outside of my immediate family. My friends and I would use applications like Netflix Party in order to still do things together virtually, although the whole summer left me feeling very isolated.

As a result of the pandemic, my senior year was far from traditional. Everyone would tell you that senior year was going to be the best year of high school, but it was by far the worst. We started with everything still being online, and I found that my peers and I struggled to stay motivated. The lack of structure and routine took a toll on me, and I turned in many assignments late, which is something I had never done in the past. It got to a point where it was so normal to turn things in months after the deadline that my teachers had to start being stricter about it, although it was hard to enforce as the school did not want to act against people's grades. My high school eventually adopted the hybrid format, where you could go in‐person 2 days a week. These days were assigned in order to not have too many people in the building at once. I opted into this format as I was so desperate to have any kind of social interaction that was not through a computer screen, even though a year ago I would have jumped at the chance of being able to stay home from school. Unlike the class of 2020, we did have an in‐person graduation on a football field, which I was very grateful for. We also were able to have a prom outside that was planned by the parents of students at my school. While I was able to have these hallmark high school experiences, how untraditional they were made me feel like I had missed out on a Disney Channel high school experience.

I feel that the long‐term effects of COVID‐19 on the youth are most prevalent in education and the economy. During the pandemic screens became abruptly integrated into schools as all assignments were moved online. I think this has its benefits, but I also feel that there are now too many distractions in the classroom, as having so many devices makes it easy to tune out from what is going on in class. Additionally, I think it is much more normalized for students in middle and high school to turn in assignments late. My brother, who is a high school student, has consistently turned in many assignments late over the past couple years with little to no repercussions. I have noticed his school system and others becoming stricter with this, but I believe this hurts students in the long run as colleges are much less accepting of late work. Furthermore, now most assignments are online, which in a lot of cases has replaced discussion‐based learning. I feel that there is a big difference posting on an online discussion board versus having an in‐person discussion, with the latter being more valuable for students. I believe that in‐person discussions are beneficial as they allow students to socialize and bounce ideas off each other, which is something that online learning tends to lack. Students tend to be more engaged, indulge in collaborative critical thinking with improved understanding and take away more from an in‐person discussion.

In regard to the economy, the inflation that occurred during the pandemic skyrocketed prices for groceries and housing in a way that hadn't been seen in years. I think this worries people my age, especially those that are just graduating college and looking for full‐time jobs. While inflation is a normal phenomenon, it feels that the pandemic has contributed significantly to the rising costs of goods across the United States. I would like to see proactive legislation combating inflation costs or capping rent prices, as the fear of not being able to afford to live is a big stressor for many people my age, especially since the wages for jobs have not increased much. Combined with the skyrocketing cost of higher education, a lot of people my age contemplate whether or not it is even worth it to get a college degree if everything keeps becoming more and more expensive.

Additionally, the pandemic appeared to contribute to an increase in social media usage, which has been problematic for my generation. I think it was beneficial for me at the time as it allowed me to connect with my friends in a way I would not have been able to otherwise, but now it feels that we rely on social media for everything, including our news and information. There was a lot of misinformation spread during COVID about vaccines, and I feel that ever since the pandemic the spreading of misinformation online has become a big problem. As someone who studies biology, it concerns me that so many things are spread on social media with little to no scientific evidence. I would like to see more of an effort on the social media companies themselves to fact check information.

Lastly, the forced isolation during the pandemic, while making me closer to my immediate family, also brought about feelings of resentment. Before the pandemic, I viewed time with my family as a privilege. During the pandemic, since I was no longer able to see my friends, I felt forced to spend time with my immediate family. There were parts of this I appreciated, although I think it increased my desire to move away for college even more, especially since I was a teenager who just wanted independence. During the pandemic I got a job as a delivery driver, and while normally that would not be something I enjoy, I was so grateful for it as that it was my only way to leave the house and interact with people outside of my family. As a student in college now, I miss being able to see my family every day and go home whenever I can, and I appreciate that seeing my family feels more like a privilege again than a chore. Thankfully I have a good relationship with my family, but for others who don't feel that way I could see how the pandemic would take a much greater toll on their mental health.

## CONCLUSION

COVID‐19 impacted my life in a variety of ways, including social contact with friends, a sharp transition from offline to online classes, cost of living, social media use, and my relationship with my family. While these impacts were inevitable, I believe it is important to study these impacts and how they could be impacting the youth currently and in the future. Most importantly, I believe more can be done legislatively to combat the ongoing long‐term effects of the pandemic, specifically within education and corporate greed which resulted in inflation. Without acknowledging the realities of the pandemic and its effects, my generation will not be set up to succeed in the future.

## FUNDING INFORMATION

The author declares no funding.

## CONFLICT OF INTEREST STATEMENT

The author declares no conflicts of interest.

## CONSENT

Not applicable.

## Data Availability

Data sharing not applicable to this article as no datasets were generated or analyzed during the current study.

